# Comparative Analysis of Passive Movement During Robot-Assisted and Therapist-Led Rehabilitation Exercises

**DOI:** 10.3390/s25175334

**Published:** 2025-08-28

**Authors:** Iwona Chuchnowska, Jolanta Mikulska, Michał Burkacki, Marta Chmura, Miłosz Chrzan, Jan Kalinowski, Sławomir Suchoń, Marek Ples, Mariusz Sobiech, Piotr Szaflik, Hanna Zadoń, Beniamin Watoła

**Affiliations:** 1 Faculty of Biomedical Engineering, Silesian University of Technology, Roosevelta 40, 41-800 Zabrze, Poland; michal.burkacki@polsl.pl (M.B.); marta.chmura@polsl.pl (M.C.); milosz.chrzan@polsl.pl (M.C.); jankali123@student.polsl.pl (J.K.); marek.ples@polsl.pl (M.P.); piotr.szaflik@polsl.pl (P.S.); hanna.zadon@polsl.pl (H.Z.); beniwat159@student.polsl.pl (B.W.); 2 Special Educational and Training Centre in Ziemięcice, 120 Mikulczycka Street, 42-675 Ziemięcice, Poland; j.mikulska@rfpn.org; 3 Łukasiewicz Research Network—Krakow Institute of Technology, Centre for Medical Engineering, Roosevelta 118, 41-800 Zabrze, Poland

**Keywords:** rehabilitation, passive exercises, UR10e, Noraxon Ultium Motion, upper limb, manual rehabilitation, motion analysis

## Abstract

The growing number of patients in need of rehabilitation, largely due to an aging population and the increasing incidence of strokes, drives the search for more effective therapeutic methods. Stroke remains a leading cause of adult disability, increasing demand for rehabilitation services. Robotic-assisted therapy presents a promising solution by offering precision and repeatability, complementing traditional methods. This study compared traditional rehabilitation led by a physiotherapist with robotic-assisted therapy using the UR10e robot. The research consisted of two stages: in the first, a physiotherapist guided passive upper limb movements, and in the second, the same movements were replicated by the UR10e robot with a specialized adapter for arm positioning. Movements were measured using the Noraxon Ultium Motion system, analyzing flexion, extension, and rotation angles at the shoulder and elbow joints.

## 1. Introduction

Rehabilitation plays a key role in the recovery process of patients after various types of injury, surgery, or neurological diseases. It is a comprehensive process that aims to restore physical function, enable patients to be independent, and improve their quality of life. Research and development of assistive technologies for rehabilitation, including the field of biomedical engineering, play a key role in improving rehabilitation processes and their capabilities. Automated rehabilitation equipment provides timely and effective rehabilitation training, which is key to accelerating recovery [1,2]. Due to advances in technology, rehabilitation therapies are becoming increasingly advanced and effective, opening new possibilities for patients. Today, different types of robots can play an important role in the context of rehabilitation, bringing many benefits to patients and therapy professionals [3,4].

There are a number of conditions for which the use of robots may be particularly effective. One potential application could be therapies for patients after stroke, with spinal cord injuries, established multiple sclerosis, Parkinson’s disease or post-traumatic brain injuries, allowing for regaining or improving motor function, thus improving quality of life [5,6]. As the number of stroke patients increases, so does the demand for rehabilitation training. Robot-assisted training is expected to play a key role in meeting this demand [7,8].

In the context of the use of robots to support rehabilitation, there are many potential benefits and opportunities to use these advanced technologies [9]. Robots can be used to deliver therapy in both clinical and home settings, allowing patients to access effective rehabilitation care anywhere, anytime. In addition, robots offer precise control over the movements performed and the ability to monitor therapeutic progress, resulting in a more personalized and tailored therapy [10,11].

The advantages of robotic therapy are numerous and contribute significantly to the effectiveness of this type of intervention. Repeatability and the ability to plan the exact number of repetitions per unit of time translate into greater control of movements and the entire exercise, which can result in accelerating the recovery process. Furthermore, by supporting specific movements of body parts, rehabilitation robots contribute to reducing the workload of rehabilitation workers. As a result of the advantages above, an approach that assumes the presence of robots in rehabilitation processes can lead to better and longer-lasting therapeutic effects [12].

The use of robots in rehabilitation represents a promising prospect for improving the care of patients with various neurological and orthopaedic conditions. Research and technology development in this field is key to improving therapeutic methods and providing patients with more effective and personalized care. With innovative solutions such as rehabilitation robots, it is possible to speed up the recovery process and improve the quality of life of those in need of rehabilitation [13].

The research methodology was designed to enable a detailed comparative analysis of joint angles, range of motion, and movement trajectory fidelity between manually guided and robot-assisted limb actuation. The primary objective was to evaluate the capability of the robotic system to replicate or enhance movement patterns typically executed by a physiotherapist, rather than to assess therapeutic efficacy or patient recovery outcomes.

The primary aim of this study was to conduct a direct, quantitative comparison between traditional rehabilitation performed by an experienced physiotherapist and robotic-assisted therapy using the UR10e robotic system. By analyzing identical passive upper limb movements executed in two conditions—manually by the physiotherapist and mechanically by the robot—we sought to determine differences in movement repeatability, precision, and range of motion at the joints.

The need for such a comparison arises from the growing integration of robotic systems into rehabilitation practice, where objective data are required to evaluate whether robotic-assisted therapy can match or exceed the consistency and accuracy of manual techniques. Establishing this baseline under controlled conditions with a healthy participant eliminates variability introduced by pathology and allows for a clear assessment of the mechanical performance and potential advantages of robotic execution before proceeding to clinical trials with patient populations.

## 2. Materials and Methods

The experimental evaluation was conducted on an adult male participant (23.5 years old). The subject was in good general health and reported no history of upper limb injuries, musculoskeletal disorders, or neurological conditions that could influence upper limb mobility. He was right-handed and had no prior exposure to robotic rehabilitation technologies. His participation aimed to establish a normative dataset for healthy upper limb kinematics, which will serve as a control reference in subsequent studies involving individuals with motor impairments. This man was chosen to reflect a standard baseline of healthy joint function and motor performance. Prior to participation, all the individuals provided informed consent, and the study protocol was approved in accordance with the ethical principles of the Declaration of Helsinki.

We deliberately selected a healthy participant. Patients with injuries or neurological impairments often present highly variable and unpredictable movement patterns, which could make it more difficult to isolate the effects of the movement execution method (robot vs. physiotherapist). Testing on a healthy subject allows us to determine the robot’s movement precision, repeatability, and control under optimal biomechanical conditions before introducing the additional variability caused by pathological movement. Using a healthy participant minimizes the risk of adverse events during early-stage testing, while allowing both the physiotherapist and the robot to execute full ranges of motion without clinical restrictions. In the absence of compensatory strategies or pain-related movement limitations, it is easier to attribute observed differences directly to the method of execution rather than to individual patient-specific impairments.

**Figure 1:**
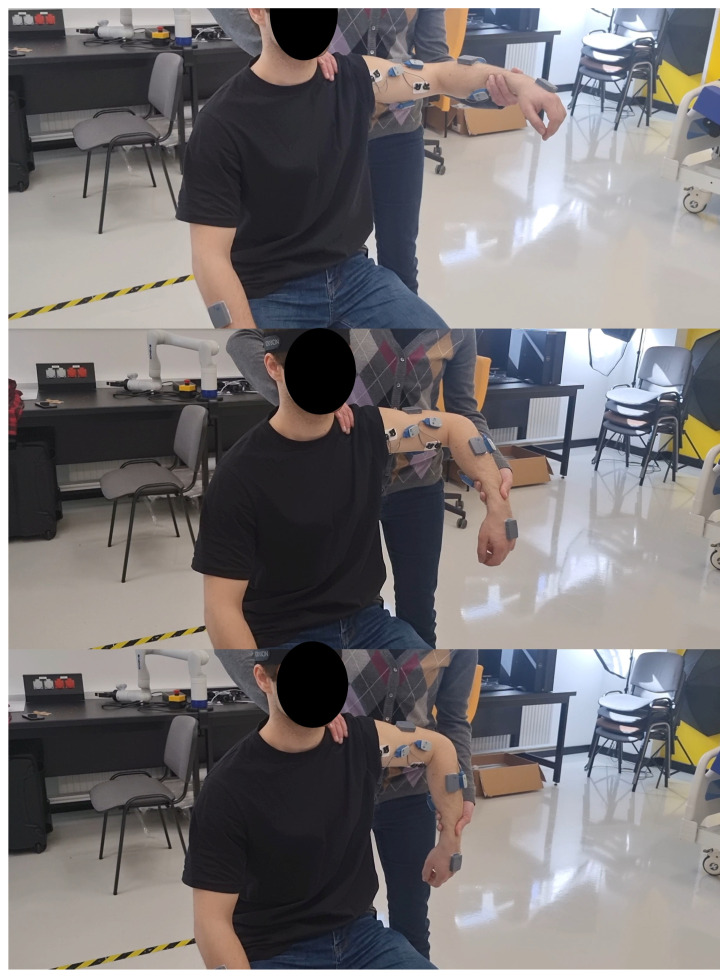
Example of a rehabilitation exercise led by a physiotherapist.

Manual therapy sessions were conducted by a Master of Physiotherapy, certified in the NDT-Bobath concept and the Vojta method, with expert-level knowledge and extensive experience in rehabilitation (Figure 1).

**Figure 2:**
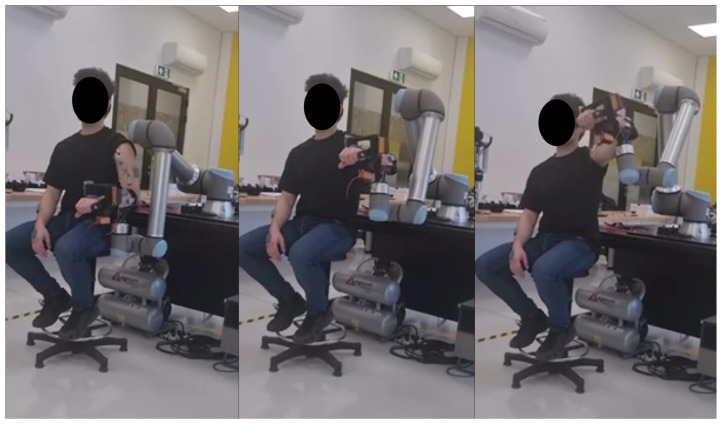
Example of a rehabilitation exercise performed with a UR10e robot with a special tip.

Experimental studies of upper limb motility were carried out using a UR10e robot and a specially designed tip (Figure 2). The robot was responsible for driving the shoulder and elbow joints, while the prepared robotic tip allowed movements at the wrist joints. The UR10e robot used in this study is an industrial collaborative robotic arm, primarily designed for high-precision and repeatable movements in manufacturing and automation contexts, rather than specifically for rehabilitation purposes. This distinguishes it from many rehabilitation-focused robotic systems, which often integrate specialized sensors, adaptive control algorithms, and patient-engagement interfaces to adjust assistance in real time based on patient feedback or physiological signals. Unlike robots equipped with features such as adaptive learning, intention detection via EMG, or “learning-by-demonstration” approaches, the UR10e operates based on predefined trajectories and does not autonomously adapt its movement to changes in patient condition during the session. The advantages of using the UR10e in this study include its repeatability, ease of programming, and modularity for adapting to different exercises. However, its lack of built-in clinical adaptation features means that it relies entirely on careful programming and mechanical setup to replicate physiotherapist-guided movements.

The Noraxon MyoMotion system (Noraxon U.S.A., Inc., Scottsdale, AZ, USA), which includes nine sensors, was used to measure the data. These were positioned as follows: one sensor on the forehead, two on the spine (one at the sacral region and one at the cervical region), and three sensors for each upper limb (hand, forearm, and shoulder) [14].

Before the study, in collaboration with the physiotherapist, it was determined what movements would be performed and how many repetitions would be required, and the feasibility of performing them on the robot was confirmed. Passive exercises performed by the physiotherapist were selected to work through the full range of motion of the given joints and activate the muscles at their maximum potential. Passive exercises performed by the physiotherapist are most similar to the movements performed by the robot, which reduces statistical error and makes the studies more reliable. Exercises performed by the physiotherapist and the robot were performed in the same starting position. Passive exercises were performed by the physiotherapist and the robot according to the specified procedures, starting with the proximal shoulder joints and ending with the distal joints in the same order, which was important in obtaining results with less measurement error. The upper limb motorization test consisted of two stages, where, in the first stage, the physiotherapist was responsible for the motorization of the limb. The physiotherapist set the hand in passive motion by performing predetermined rehabilitation exercises. In the second part of the study, this task was performed by a specially prepared robot. Each successive movement with the UR10e had to be programmed first by determining successive positions for the robot. The positioning of the robot arm was performed with a high degree of care in order to replicate the physiotherapist’s work as closely as possible. Apart from this difference, both steps followed the same procedure. A calibration of the Noraxon system was performed before each movement, and each specific movement was performed at least twice to ensure that it was performed correctly in the subsequent stages of the study, thus eliminating potential errors. The UR10e has a reach of 1300 mm, a payload capacity of 12.5 kg, and six degrees of freedom, with 360 degrees of rotation for each component. We used the moveJ command, which performs trajectory interpolation in joint space. Using this specific command is a trade-off: on one hand, the robot’s motion is faster and smoother; on the other hand, the TCP path is less precise than with linear motion (e.g., moveL command) due to the blend radius, which introduces a relatively small deviation from the straight, shortest path between waypoints. With this command, the control mode corresponds to position control, where force feedback is used only for safety purposes—exceeding a predefined force threshold triggers an emergency stop. The robot also has an operation view panel so that the progress of the examination could be monitored and interrupted when necessary, and the speed of rotation of the robotic arm segments could be manipulated. The attached handpiece has the ability to drive the patient’s hand at the wrist joints by performing 180-degree rotations relative to the sagittal plane and transverse plane. Robotic tip servos operate independently of the UR10e robot using a laboratory power supply. The power supply operated at 4.4 V, since this was the voltage at which the drives performed the smoothest hand movements.

The rehabilitation movements that were performed during the study were shoulder joint inversion/adduction, wrist joint inversion/adduction, external and internal rotation, shoulder joint horizontal extension, shoulder joint horizontal flexion, shoulder joint flexion/extension, and elbow joint flexion/extension with pronation.

## 3. Results

### 3.1. Flexion and Straightening at the Wrist Joint (Figure 3)

**Figure 3:**
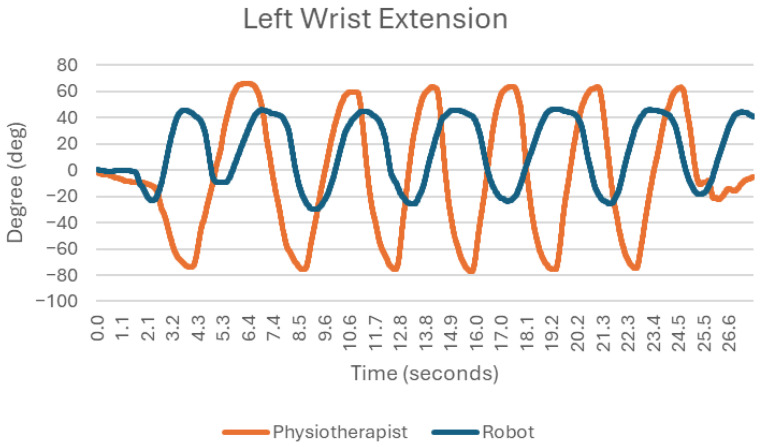
Chart of left wrist extension.

The chart below (Figure 3) illustrates a flexion and straightening exercise at the wrist joint, comparing the movement patterns performed by a physiotherapist and a robot. The exercise involves repetitive bending (flexion) and extending (straightening) of the wrist over a period of approximately 26.6 s. The physiotherapist’s motion (orange line) exhibits a greater range of movement, fluctuating between −80° (deep flexion) and +70° (full extension). This highlights the physiotherapist’s ability to achieve maximum range in both directions of wrist movement. The robot’s motion (blue line), on the other hand, is more constrained, staying within a range of −40° to +40°, indicating a more limited ability to mimic extreme positions of wrist flexion and extension. Both the physiotherapist and the robot maintain a similar periodic rhythm, performing several cycles of flexion and extension over time. However, the robot exhibits slightly less frequent oscillations, likely due to its smaller range of motion and more deliberate pacing. There is a visible lag or mismatch between the peaks and troughs of the two lines. This indicates that the robot, while following the general rhythm of the physiotherapist’s movements, does not perfectly replicate the timing or speed of transitions between flexion and extension. The physiotherapist’s movement represents the natural biomechanics of wrist exercises, with larger and variable movements reflecting both flexibility and strength. This variability is beneficial in rehabilitation settings, as it can adapt to patient needs, target different muscle groups, and accommodate joint limitations. The robot’s movement is more controlled and limited, which could be advantageous for tasks requiring high precision and safety, such as assisting a patient with limited mobility or preventing overextension. However, the reduced range and smooth, repetitive nature may limit the robot’s ability to fully replicate the natural dynamics of human joint movement, potentially reducing its effectiveness in certain rehabilitation scenarios.

These discrepancies primarily reflect the mechanical limitations of the robotic system and the initial programming of joint positions, which constrained its ability to reproduce the full ranges of motion achieved by the physiotherapist.

### 3.2. Reversal and Adduction at the Wrist Joint (Figure 4)

**Figure 4:**
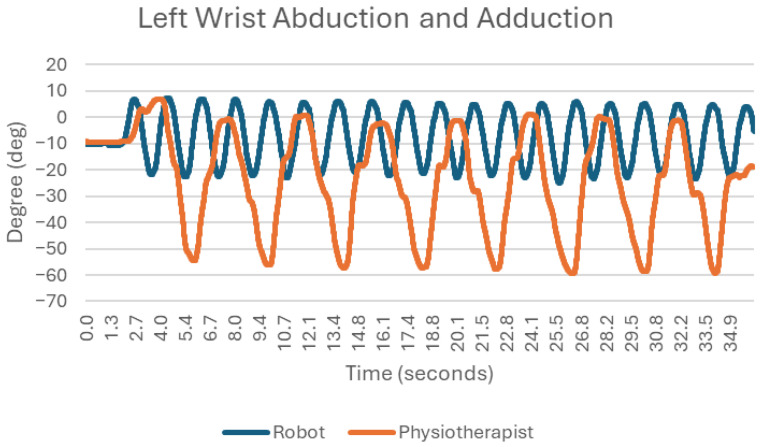
Chart of left wrist abduction and adduction.

Movement of the wrist in the frontal plane actually describes a situation very similar to flexion and extension of the wrist. There is little variation in the angle between successive movements. A high degree of repetition is apparent, but in both cases, some disruption of the movement is evident, manifested by regular, very short pauses (Figure 4).

### 3.3. Flexion and Extension with Pronation in the Elbow Joint (Figure 5)

In both cases, the accuracy of movements during flexion and extrusion at the elbow joint is very similar. Angle changes occur very smoothly, and repetition of movements is strongly noticeable. Bending and straightening in cooperation with the robot manifests itself in a much smaller range of movement. This is a difference of almost 60 degrees compared to exercise with the physiotherapist, where the flexion angle is just over 100 degrees [15,16].

### 3.4. Horizontal Extension of the Upper Limb

**Figure 6:**
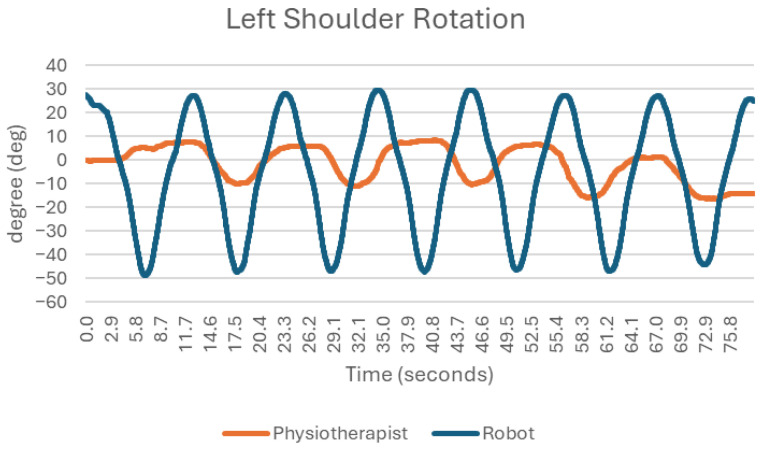
Chart of left shoulder rotation.

**Figure 7:**
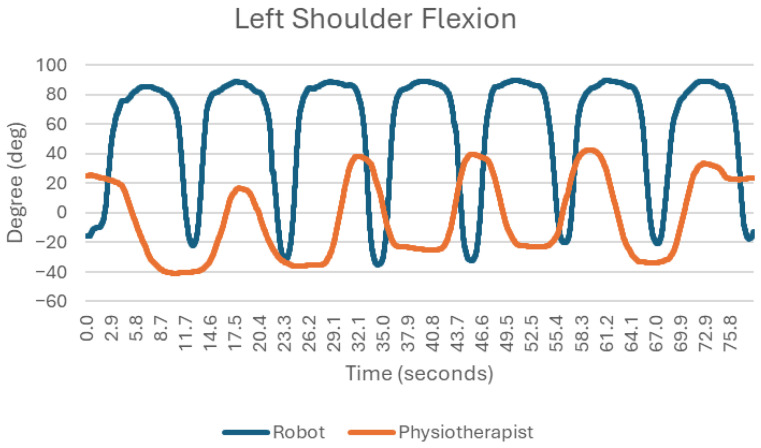
Chart of left shoulder flexion.

In this exercise, the robot demonstrated smoother and more repeatable shoulder joint movements compared with the physiotherapist. When performed manually, the rotation angles exhibited lower repeatability (Figure 6), with relatively small ranges of motion (on average, angle changes of about 20 degrees) and deviations of approximately 5 degrees. In contrast, the robot maintained smooth movements with a high degree of accuracy and repeatability. Much larger ranges of movement are also achieved, as the limb flexes by approximately 80 degrees from the input position. The flexion/extension movements at the shoulder joint during this exercise do not occur in the horizontal plane but are important in the context of this movement, as they can affect its stability and direction (Figure 7). When analyzing the bending and straightening movements, it is easy to conclude that the robot performed better. The cyclicity and fluidity of the movement is much more apparent than in the exercise with the physiotherapist. The maximum angle excursions during successive movements using the traditional method are very different (once there is a flexion of 57 degrees and in the next movement, there is already 75 degrees).

### 3.5. External Rotation (Figure 8)

External rotation of the shoulder joint is quite smooth in both cases; there is repeatability of movement, and the maximum angle excursions in successive movements are at a similar level (exercise with the robot: maximum excursion of about 35 degrees, exercise with the physiotherapist: about 30 degrees). Although the movements were broadly similar in both cases, the robot demonstrated smoother angle transitions and less abrupt increases during external rotation compared with the physiotherapist.

### 3.6. Internal Rotation of the Shoulder Joint (Figure 9)

The internal rotation performed with the robot looks more stable than exercise in the traditional way.

### 3.7. Flexion and Straightening of the Shoulder Joint

**Figure 10:**
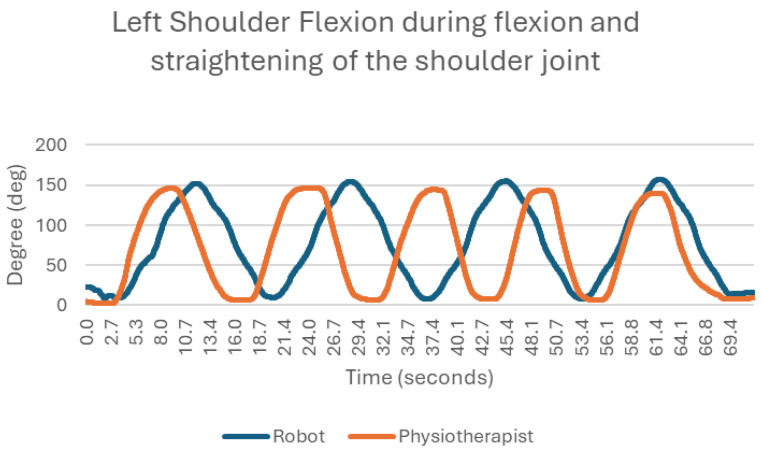
Chart of left shoulder flexion during flexion and straightening of the shoulder joint.

Flexion of the shoulder joint is correct in both cases [17]—the movements are smooth, the maximum excursions of the limb are practically identical (approx. 150 degrees), and there is a high degree of repeatability (Figure 10). However, there are some differences between the two. The exercise with the rehabilitation robot is more stable; this means that the transition moments between flexion/extension and straightening/bending movements are more uniform. In other words, the duration of each successive movement is very similar to the previous.

### 3.8. Reversal and Adduction at the Shoulder Joint (Figure 11)

Inversion and adduction of the limb when tested with the robot showed much better repeatability. The durations of the individual movements of inversion and adduction are uniform, whereas when observing the changes in angle in the examination with the traditional method, no such relationship can be seen. When observing the movements carried out by the physiotherapist, it can be seen that they differ in the maximum angles of inversion and adduction of the limb (first repetition of inversion, about 150 degrees, fourth repetition, about 130 degrees). In the robotic test, the variation between successive limb pivots was smaller, with the values remaining consistently close to 125 degrees.

**Figure 11:**
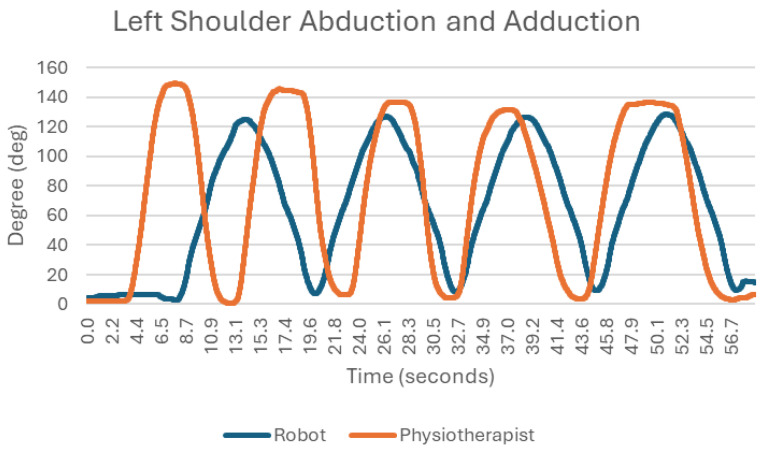
Chart of left shoulder abduction and adduction.

Table 1 presents a quantitative comparison of the range of motion (ROM) achieved during passive upper limb exercises performed by a physiotherapist and the UR10e robot. In most cases, the robot demonstrated a smaller ROM compared to the physiotherapist; for example, there was only 43% ROM coverage during elbow flexion/extension and approximately 50–54% during wrist movements. This indicates that while the robot can execute basic motion patterns, its mechanical limitations may hinder replication of the full anatomical range, especially in complex joints or multi-planar tasks.

Interestingly, in some movements, the robot achieved a greater ROM than the physiotherapist, such as 149% in horizontal shoulder flexion and 240% in shoulder rotation. These results suggest a high level of repeatability and trajectory control, particularly for planar, programmable motions. In contrast, the physiotherapist-led exercises exhibited greater flexibility and adaptability—especially evident in internal shoulder rotation—reflecting the human ability to adjust movements in real time based on joint resistance and patient feedback.

Overall, Table 1 reinforces that robotic systems excel in repeatability and stability of movements, whereas physiotherapists remain indispensable in tasks requiring precision, joint-specific sensitivity, and patient-specific adjustments. These findings support the rationale for a hybrid rehabilitation approach that leverages both robotic precision and human expertise.

Table 2 summarizes the average durations and standard deviations of individual rehabilitation movements performed by the physiotherapist and the robot. In general, the robot performed most movements with lower standard deviations compared to the physiotherapist, indicating greater temporal consistency. However, certain movements, such as horizontal extension of the upper limb and external rotation, showed slightly higher variability for the robot. One possible explanation for this pattern lies in the mechanical and control characteristics of the robotic system. The UR10e robot followed pre-programmed trajectories, but for movements involving complex multi-joint coordination (such as horizontal shoulder extension or rotational movements), small variations in load distribution and mechanical compliance of the arm adapter may have led to minor timing inconsistencies. Additionally, these particular movements required larger or more intricate ranges of motion at the shoulder, which could amplify small deviations in the control loop or mechanical backlash, resulting in higher variability.

Despite these exceptions, the overall trend across the dataset still supports the conclusion that robotic execution tends to be more temporally consistent while also highlighting that for certain complex or wide-range movements, further refinement of trajectory control could improve performance. This reflects the robot’s mechanical precision and ability to execute pre-programmed motion patterns with high repeatability.

In most cases, the average movement durations between robot and physiotherapist were comparable, with some deviations. For example, in shoulder internal rotation, the robot executed the movement much faster (9.77 s vs. 14.48 s, 148% difference), whereas in wrist abduction/adduction, the robot was significantly slower (1.80 s vs. 3.95 s, 220% difference). This variability likely stems from the robot’s fixed motion profile, which does not dynamically adapt to joint resistance or patient feedback, unlike the physiotherapist who adjusts speed intuitively during treatment.

Overall, these findings underscore that robotic systems offer high temporal repeatability, which is valuable for standardizing rehabilitation exercises and tracking progress. However, the human-led therapy remains superior in responsive timing, which may be critical in adapting exercises to individual patient needs and comfort.

## 4. Discussion

The obtained results confirm that the use of the UR10e robot allows for movements to be performed with greater repeatability and precision compared to manual therapy conducted by a physiotherapist. This is particularly evident in exercises requiring high accuracy, such as horizontal abduction of the upper limb or shoulder joint rotations. In these movements, the maximum angular deviations between repetitions were observed to be less than 5°, indicating a high level of stability and control over the movement trajectory. Such high repeatability is especially important in the context of tracking patient progress and objectively evaluating the effectiveness of therapy.

On the other hand, in some exercises, the temporal smoothness and repeatability of movements generated by the robot were comparable to those performed by the physiotherapist, despite clear differences in range of motion. This suggests that, in many cases, the experience and skills of the therapist can achieve a comparable level of effectiveness. Therefore, it is important to avoid an extreme standpoint in which robotic therapy is regarded as a complete substitute for human-delivered therapy.

It should be emphasized that the discrepancies observed in wrist and elbow flexion/extension were attributable not only to the programming of the initial positions but also to the inherent design of the UR10e robot. This system was originally developed for industrial applications, prioritizing precision and repeatability rather than biomechanical adaptability. In contrast to a physiotherapist, who can dynamically adjust force, angle, and timing in real time in response to limb resistance, the robot follows predefined, rigid trajectories. This limitation becomes particularly evident during complex, multi-joint movements, where a physiotherapist can achieve full ranges of motion that the robot is mechanically unable to reproduce.

It should also be noted that the observed discrepancies in wrist and elbow flexion/extension were not only attributable to the programming of the initial positions but also to the inherent design of the UR10e robot. This device was originally developed for industrial applications, with an emphasis on precision and repeatability, but without built-in biomechanical adaptability.

Another important factor contributing to the observed discrepancies is the absence of proximal stabilization during robot-assisted movements. When a physiotherapist performs passive mobilization of a specific joint, they are able to stabilize adjacent joints so that the motion occurs almost exclusively in the targeted joint (e.g., the wrist). In contrast, the UR10e was attached only at a single point—the hand. As a result, during wrist mobilization, the absence of stabilization allowed compensatory displacements in the elbow and shoulder. Given that wrist movements have relatively small amplitudes, even minor shifts in proximal joints significantly influenced the effective range of motion recorded at the wrist. By comparison, shoulder movements in this study followed simpler trajectories with larger amplitudes, which facilitated their accurate and repeatable reproduction by the robot despite the lack of proximal stabilization. Interestingly, in some movements, the robot achieved a greater range of motion than the physiotherapist, such as 149% in horizontal shoulder flexion and 240% in shoulder rotation. This outcome can be explained by the mechanical design of the UR10e, which allowed it to follow programmed trajectories across the full angular capacity of its joints. In contrast, manual execution by the physiotherapist is subject to both anatomical constraints of the participant and the ergonomics of manipulating the limb—movements at the shoulder, in particular, require higher effort and less favorable leverage compared to the elbow or wrist. Furthermore, clinical practice does not typically emphasize achieving maximal ROM at all costs, but rather focuses on ranges that are safe, functional, and physiologically relevant. Importantly, in our study, all the robot-assisted movements remained within physiologically safe limits. Thus, the observed larger ROM in certain tasks reflects differences in biomechanics and task execution rather than a clinical advantage of the robot.

A key advantage of robotic systems lies in the elimination of human-related factors, such as errors and subjective assessments. The robot operates based on programmed parameters, ensuring uniformity of exercises and standardization of conditions, thereby enabling precise monitoring of the rehabilitation process. Furthermore, the use of such systems can significantly reduce the workload of physiotherapists in repetitive and time-consuming exercises, allowing them to focus on areas requiring manual intervention, diagnostics, or individualized therapeutic approaches.

One of the main advantages of using a robot is the elimination of subjectivity that can affect the results of human measurements [18,19]. Robots provide objective and repeatable measurements, which are particularly important in the context of monitoring rehabilitation progress and assessing the effectiveness of therapy [20,21]. Additionally, the automation of measurements can significantly increase the efficiency of clinical work, allowing physical therapists to focus on other aspects of therapy.

When comparing the two methods, it is also important to highlight the role of physical therapists. Their experience and ability to adapt techniques to the individual needs of patients is irreplaceable. Integrating robotic technology into the work of physiotherapists can lead to better rehabilitation outcomes, combining precision and repeatable measurements with a personalized therapeutic approach [22]. More accurate and repeatable measurements can lead to more personalized and effective therapeutic plans, which is beneficial for both patients and therapists [23,24].

One limitation of the present study is that the manual therapy condition was performed exclusively by a single physiotherapist. Although this therapist was a highly specialized expert with extensive clinical experience and recognized competence in rehabilitation techniques, the use of only one operator inherently limits the generalizability of the findings to the broader population of physiotherapists.

The primary reason for selecting a single therapist was to minimize inter-operator variability. Manual therapy is inherently influenced by individual technique, motor control, and therapeutic style. Involving multiple therapists could have introduced variability unrelated to the core research question, namely, the comparison between human-delivered and robot-assisted rehabilitation. By standardizing the manual condition to one expert, this study ensured consistency in movement execution, timing, and applied forces, which, in turn, allowed for a more controlled and direct comparison with the robotic system.

However, this methodological choice also means that the results reflect the performance and style of a single highly skilled practitioner rather than capturing the range of variation that might exist among therapists with different experience levels, training backgrounds, or treatment approaches. In real-world rehabilitation settings, the variability in therapist skill and technique may be greater, and therefore the differences observed between manual and robotic therapy could differ from those reported here.

Future studies will expand the sample of therapists, including individuals with varying levels of experience, to better assess how robotic systems compare to manual therapy across a broader range of clinical practice. This would allow for a more comprehensive evaluation of both the potential and the limitations of robotic rehabilitation in diverse therapeutic contexts.

Robots can deliver consistent, repetitive movements over extended periods, allowing patients to benefit from longer and more intensive therapy sessions without fatigue on the part of the therapist. Robotic systems ensure high repeatability in movement execution, which is particularly beneficial for neuromotor re-education and minimizing compensatory patterns. Robots can collect detailed data on patient performance, enabling personalized adjustments to therapy and providing clear metrics for progress evaluation. Many robotic systems incorporate interactive elements, such as gamification or real-time feedback, which can improve patient motivation and adherence to therapy. Robotic devices can be programmed to operate within safe ranges of motion and adapt to the patient’s current capabilities, reducing the risk of injury and promoting gradual improvement.

At the same time, caution must be exercised when interpreting the results. This study was conducted under experimental conditions, with a limited range of movements and only one participant. Future research should analyse the effectiveness of robotic therapy in a long-term perspective and with the participation of diverse patient groups, such as individuals post-stroke or with neurological disorders or orthopedic injuries.

We used a single participant in the present study. A kinematic analysis of one individual cannot be considered a normative dataset for healthy upper limb motion. However, the primary objective of this work was not to establish normative values, but rather to conduct a controlled, exploratory comparison between manual rehabilitation performed by an experienced physiotherapist and movements executed by the UR10e robotic system.

Including only one healthy participant allowed us to eliminate inter-subject variability and focus exclusively on the differences attributable to the method of movement execution rather than to anthropometric or physiological differences between participants. Variations in limb length, body mass, and motor habits could introduce confounding factors, making it more difficult to isolate the direct impact of the robotic versus manual approach. By using a single subject with anthropometric characteristics typical of young adults (mean age ~24.6 years for our student population), we can provide a stable reference point for the initial phase of the research.

It is important to note that the repeatability and trajectory control of the robotic system are determined primarily by its programmed parameters and mechanical precision and are far less sensitive to anthropometric variability than human-performed movements. This makes a single-subject protocol sufficient for identifying baseline performance differences between robot-assisted and manual execution under standardized conditions.

Future studies will include participants with a range of anthropometric profiles to evaluate whether these differences remain consistent across diverse populations.

In summary, the greatest potential of robotic therapy is revealed in situations where high repeatability, precision, and monitoring of biomechanical parameters are required. However, optimal outcomes can only be achieved through the integration of robotic work with the knowledge and experience of physiotherapists. Such a hybrid approach can not only increase therapy effectiveness but also improve its accessibility and quality within the healthcare system.

The general conclusions drawn from the current analysis are consistent with the findings presented in [14]. In that study, the authors also observed that the robot was able to successfully replicate therapist-guided upper limb movements, although with reduced joint range of motion. Furthermore, they emphasized the robot’s strong temporal repeatability, i.e., the ability to execute movement cycles with consistent timing. These observations are confirmed by the present results: while the robot’s range of motion was limited in several joints compared to the physiotherapist, it demonstrated significantly lower variability in movement durations, indicating a high level of control and consistency. This strengthens the argument for the use of robotic systems in scenarios requiring standardized, repeatable motion patterns, particularly for monitoring and data-driven evaluation of rehabilitation progress.

## 5. Conclusions

While robotic-assisted rehabilitation demonstrates clear advantages in precision, repeatability, and efficiency, it cannot fully replace the essential role of physiotherapists. The human element in therapy, particularly the ability to adjust treatment based on the patient’s needs and feedback, remains irreplaceable. Combining robotic systems with the expertise of physiotherapists offers the most effective approach, enhancing rehabilitation outcomes while maintaining personalized patient-centered care.

The UR10e robotic arm used in this study is a standard industrial robot and does not possess advanced rehabilitation-oriented functions. Specifically, it lacks integrated modules for physical workload monitoring, patient intention assessment (e.g., via EMG), performance tracking with accuracy feedback, or engagement measurement, as described in [25]. Furthermore, it does not incorporate Learning by Demonstration (LbD) or supervised learning methods based on neural networks to automatically replicate physiotherapist movements, as presented in [26].

The advantage of testing a standard, commercially available robot lies in its widespread availability, cost-effectiveness, and reproducibility of results, as well as the ability to evaluate baseline feasibility before integrating more sophisticated adaptive control and patient feedback mechanisms.

In future research, we plan to expand this study to include other robotic platforms equipped with adaptive force control, intention detection, and learning-based movement replication. Future research will be expanded to include EEG monitoring of the patient to assess neural engagement during therapy.

## Figures and Tables

**Figure 5:**
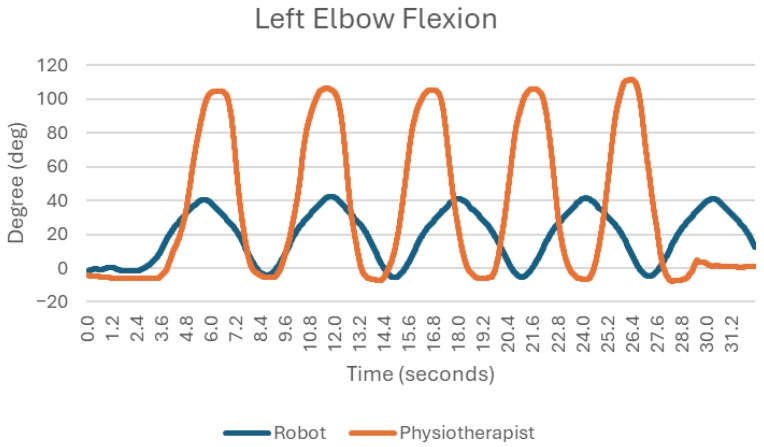
Chart of the left elbow flexion.

**Figure 8:**
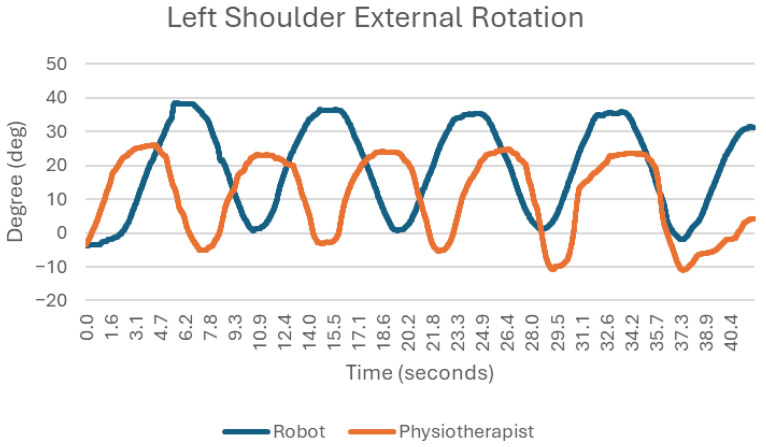
Chart of left shoulder external rotation.

**Figure 9:**
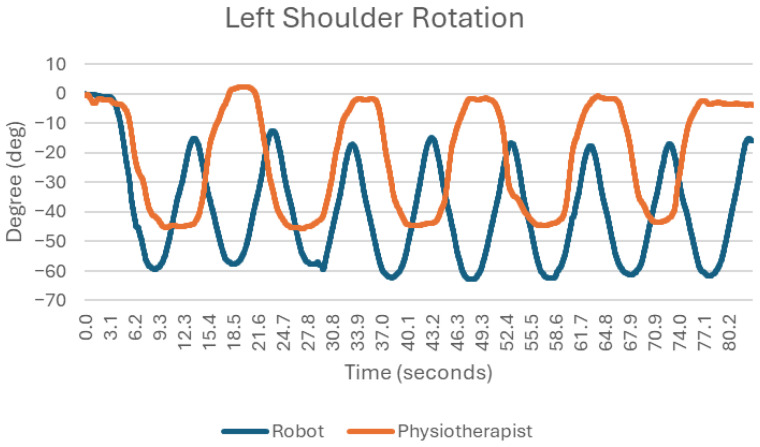
Chart of left shoulder internal rotation.

**Table 1:** Quantitative comparison of the range of motion (ROM) achieved during passive upper limb exercises performed by a physiotherapist and by the UR10e robotic device.

**Activity**	**Flexion and Straightening** **at the Wrist Joint**			**Reversal and Adduction** **at the Wrist Joint**			**Flexion and Extension with** **Pronation in the Elbow Joint**		
joint movement	left wrist extension [deg]			left wrist abduction and adduction [deg]			left elbow flexion [deg]		
	min	max	ROM	min	max	ROM	min	max	ROM
physiotherapist	−76.77	65.45	142.23	−54.72	6.21	60.92	−3.20	119.92	123.12
robot	−30.09	46.76	76.84	−20.92	9.24	30.16	1.73	51.43	53.16
percentage of coverage by robot	54%			50%			43%		
**Activity**	**Horizontal extension of the upper limb**						**External rotation**		
joint movement	left shoulder rotation [deg]			left shoulder flexion [deg]			left shoulder rotation [deg]		
	min	max	ROM	min	max	ROM	min	max	ROM
physiotherapist	−32.63	−0.47	33.11	−56.17	26.95	83.12	−15.79	21.42	37.22
robot	−58.01	21.38	79.39	−48.66	75.34	124.00	−8.09	29.67	37.76
percentage of coverage by robot	240%			149%			101%		
**Activity**	**Internal rotation** **of the shoulder joint**			**Flexion and straightening** **of the shoulder joint**			**Reversal and adduction** **at the shoulder joint**		
joint movement	left shoulder internal rotation [deg]			left shoulder flexion [deg]			left shoulder abduction and adduction [deg]		
	min	max	ROM	min	max	ROM	min	max	ROM
physiotherapist	−44.62	4.58	49.20	−12.34	144.41	156.75	−14.36	148.14	162.51
robot	−62.82	−13.21	49.61	−9.89	140.20	150.09	−15.06	111.33	126.38
percentage of coverage by robot	101%			96%			78%		

**Table 2:** Summary of the average durations and standard deviations of individual rehabilitation movements performed by the physiotherapist and the UR10e robot.

Activity	Flexion and Straightening at the Wrist Joint		Reversal and Adduction at the Wrist Joint		Flexion and Extension with Pronation in the Elbow Joint		Horizontal Extension of the Upper Limb				External Rotation		Internal Rotation of the Shoulder Joint		Flexion and Straightening of the Shoulder Joint		Reversal and Adduction at the Shoulder Joint	
**Joint Movement**	**Left Wrist Extension [s]**		**Left Wrist Abduction and Adduction [s]**		**Left Elbow Flexion [s]**		**Left Shoulder Rotation [s]**		**Left Shoulder Flexion [s]**		**Left Shoulder Rotation [s]**		**Left Shoulder Internal Rotation [s]**		**Left Shoulder Flexion [s]**		**Left Shoulder Abduction and Adduction [s]**	
	avg	std	avg	std	avg	std	avg	std	avg	std	avg	std	avg	std	avg	std	avg	std
physiotherapist	3.68	0.47	3.95	0.17	4.92	0.17	13.6	0.45	13.8	0.21	7.72	0.31	14.4	1.12	13.2	1.33	10.2	0.53
robot	3.91	0.22	1.80	0.04	6.19	0.01	11.0	0.05	11.1	0.26	9.06	0.40	9.77	0.10	16.8	0.04	12.6	0.01
difference robot vs. human	94%		220%		80%		124%		124%		85%		148%		79%		81%	

## Data Availability

The datasets used and analyzed during the current study are available from the corresponding author on reasonable request.
